# Combination immunotherapy of chlorogenic acid liposomes modified with sialic acid and PD-1 blockers effectively enhances the anti-tumor immune response and therapeutic effects

**DOI:** 10.1080/10717544.2021.1971797

**Published:** 2021-09-13

**Authors:** Xixi Li, Shunyao Zhu, Ping Yin, Shuangshuang Zhang, Juewen Xu, Qin Zhang, Senlin Shi, Ting Zhang

**Affiliations:** a School of Pharmaceutical Sciences, Zhejiang Chinese Medical University, Hangzhou, China; b School of Life Sciences, Zhejiang Chinese Medical University, Hangzhou, China

**Keywords:** Combination immunotherapy, chlorogenic acid, sialic acid, anti-PD-1 antibody, melanoma

## Abstract

Melanoma is one of the most common malignant tumors. The anti-PD-1 antibody is used for the treatment of metastatic melanoma. Treatment success is only 35–40% and a range of immune-related adverse reactions can occur. Combination of anti-PD1 antibody therapy with other oncology therapies has been attempted. Herein, we assessed whether chlorogenic acid liposomes modified with sialic acid (CA-SAL) combined with anti-PD1 antibody treatment was efficacious as immunotherapy for melanoma. CA-SAL liposomes were prepared and characterized. In a mouse model of B16F10 tumor, mice were treated with an anti-PD1 antibody, CA-SAL, or combination of CA-SAL + anti-PD1 antibody, and compared with no treatment controls. The tumor inhibition rate, tumor-associated macrophages (TAMs) phenotype, T-cell activity, and safety were investigated. We observed a significant decrease in the proportion of M2-TAMs and CD4^+^Fop3^+^ T cells, while there was a significant increase in the proportion of M1-TAMs and CD8^+^ T cells, and in the activity of T cells, and thus in the tumor inhibition rate. No significant toxicity was observed in major organs. CA-SAL and anti-PD1 Ab combination therapy presented synergistic anti-tumor activity, which enhanced the efficacy of the PD-1 checkpoint blocker in a mouse model of melanoma. In summary, combination immunotherapy of CA-SAL and anti-PD1 Ab has broad prospects in improving the therapeutic effect of melanoma, and may provide a new strategy for clinical treatment.

## Graphical Abstract

**Figure:**
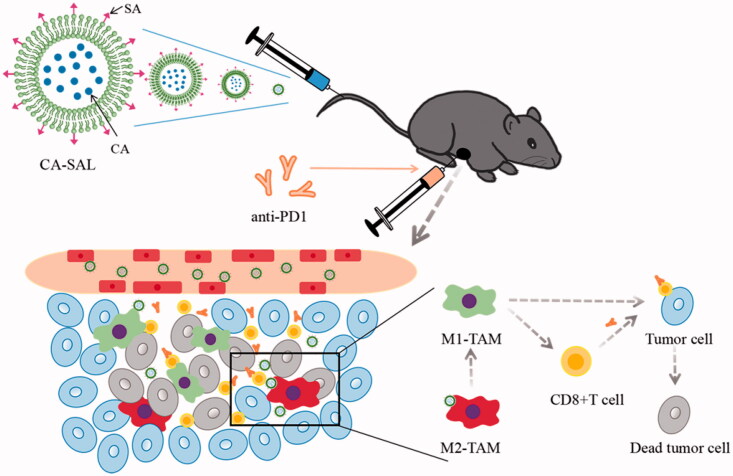

## 1. Introduction

Cancer remains one of the most pressing issues affecting human health. To date, nonsurgical treatment of cancer, such as conventional chemotherapy and radiotherapy, were not methods that could produce completely satisfactory treatment results (Burugu et al., 2018). Melanoma is one of the most common malignant tumors and is also one of the most rapidly increasing malignancies (Bomar et al., 2019) and is insensitive to chemotherapy or radiotherapy (Duncan, 2009). In contrast, melanoma restores anti-tumor immunity in the tumor microenvironment (TME). Immunotherapy is a very promising form of cancer treatment, which produces a long-lasting immune effect (Marshall & Djamgoz, 2018). The interaction of anti-programmed death protein 1 (PD-1) and anti-programmed death protein ligand 1 (PD-L1) is the mechanisms through which immunogenic tumors escape the immune response. Blocking this interaction can re-activate the body's immunity to the tumor (Tomita et al., 2020). A sustained immune response and effective immune memory may also keep tumor growth under constant control (Zhang et al., 2016). Anti-PD-1 antibody (anti-PD1 Ab) exerts long-term antitumor effects on melanoma cells and has been widely used to treat metastatic melanoma (Queirolo et al., 2019). Anti-PD1 Ab can induce an anti-tumor immune response by activating the patient's immune system (Pardoll, 2012); however, anti-PD1 Abs are only 35–40% effective in melanoma patients, and the majority of patients develop resistance (Tumeh et al., 2014; Puzanov et al., 2020). How to increase the number of patients benefiting from immunotherapy is a research hotspot (Ji et al., 2020). Combination therapy is a new approach to overcoming this problem and is crucial to achieving complete remission and cure for cancer patients (Mahoney et al., 2015). Studies have shown that combined immune checkpoint blockade therapy further enhances the immune response, enhances the efficacy of preventing and treating B16F10 tumors, and clinically inhibits B16F10 tumor metastasis and recurrence (Fan et al., 2020). The TME includes tumor-associated macrophages (TAMs), myeloid-derived suppressor cells, tumor-associated neutrophils, and myeloid dendritic cells (Balkwill et al., 2012). TAMs are the most abundant inflammatory cells infiltrating tumors and have become an attractive target for tumor treatment due to their critical role in supporting tumor progression. Furthermore, studies have shown that the decrease of M2-TAMs can enhance the anti-tumor response mediated by T cells and improves the efficacy of immunotherapy (Hagemann et al., 2008; Jaiswal et al., 2010; Coussens et al., 2013).

TAMs play a vital role in the TME (Morrison, 2016). TAMs are believed to seriously affect the efficacy of various cancer therapies by stimulating angiogenesis, promoting tumor cell metastasis, and inhibiting anti-tumor immune response (Zhu et al., 2021). TAMs can be divided into two types: proinflammatory M1 and anti-inflammatory M2 macrophages (Morrison, 2016). The difference in the function of macrophages is strongly related to their plasticity. The functional phenotype of macrophages can be influenced by molecular regulatory components of the TME. M1 macrophages play antitumorigenic roles and promote the immune response to tumors through producing immunogenic cytokines. Therefore, effective antitumor immunotherapy may depend on the polarization of TAMs to the M1-like phenotype (De Palma and Lewis, 2013). TAMs are generally polarized toward M2-TAMs. M2-TAMs promote tumor growth, infiltration and metastasis by increasing the secretion of the immunosuppressive factors, and create a favorable microenvironment to promote tumor progression, tumor metastasis, and abnormal angiogenesis (Pathria et al., 2019). Furthermore, with the participation of tumor-derived factors, M2-TAMs inhibit T-cell-mediated immune response (De Palma and Lewis, 2013). The polarization of TAMs is reversible and modifiable. Thus, it is critical to induce the polarization of TAMs from the M2- to the M1-like phenotype.

Chlorogenic acid (CA) is a polyphenolic compound that exerts biological activities including antibacterial and anticancer activity (Zeng et al., 2021). CA has a significant inhibitory effect on liver cancer, lung cancer, glioma, and other tumors and is considered to be an effective anti-cancer agent (Huang et al., 2019). Studies have shown that in melanoma, CA can induce TAMs switching from the M2 to M1 type, which produces a series of anticancer effects (Qian et al., 2017).

Nanotechnology is a research hotspot in drug delivery systems and shows great prospects in cancer immunotherapy (Xiong et al., 2019). These drug-loading systems present low immunogenicity and prolonged circulation in the blood (Yang et al., 2020). Liposomes have a bilayer lipid structure. In addition, they have excellent biocompatibility and low toxicity and have found wide application in cancer and other conditions. Studies have shown that liposomes can inhibit the proliferation of melanoma cells, and thereby inhibit tumor growth (Castañeda-Reyes et al., 2020; Huang et al., 2020).

Sialic acid (SA) is the N- or O-substituted derivative of neuraminic acid (Schauer, 2009). SA binding receptors are highly expressed on TAMs (Nath et al., 1999). And SA-modified nanocomposites can preferentially accumulate in TAM and exert effective anti-tumor activity (Qiu et al., 2019). Therefore, strategies targeting SA epitopes provide a powerful tool for cancer treatment (Fan et al., 2017). In addition, a SA-modified vector has been constructed to target TAMs-related anti-tumor therapy (Zhou et al., 2017). Finding a drug with a specific effect on TAMs and using SA-modified nanocarriers (CA-SAL) for its delivery may be a promising cancer immunotherapy strategy (Tang et al., 2020).

At present, there are few reports on the therapeutic strategy of CA-SAL combined with PD-1 Ab. To significantly improve the antitumor efficacy of antitumor drugs, we designed a targeted liposome modified with SA, and assessed its physicochemical properties, *in vitro* release, and cellular uptake. Finally, we studied the effects and mechanisms of CA-SAL combined with anti-PD1 Ab through a series of *in vitro* and *in vivo* experiments.

## 2. Materials and methods

### 2.1. Materials

SA was purchased from CLKC Medicinal Chemistry Co., Ltd (Hubei, China), N-(3-dimethyl aminopropyl)-N-ethyl carbodiimide HCl (EDC), N-hydroxysuccinimide (NHS), dimethylformamide (DMF) were purchased from Macklin (Shanghai, China), chlorogenic acid was purchased from Qiaoyu Biotechnology Co., Ltd. (Shanghai, China), octadecylamine (ODA) was purchased from Feiyu Biotechnology Co., Ltd. (Nantong, China), hydrogenated soy phosphatidylcholine (HSPC), cholesterol (CH) were purchased from AVT Pharmaceutical Tech Co., Ltd. (Shanghai, China), anti-PD-1 (clone RMP1-14; 52.8 mg) was purchased from Bioxcell (West Lebanon, NH, USA), coumarin-6 was purchased from Yuanye Biotechnology Co., Ltd. (Shanghai, China), 1,1′-dioctadecyl-3,3,3′,3′-tetramethylindotricarbocyanine iodide (DiR) was purchased from AAT Bioquest (State of California, USA), 4′,6-diamidino-2-phenylindole stain solution (DAPI), and 4% paraformaldehyde solution were purchased from Biosharp (Shanghai, China), dimethyl sulfoxide (DMSO) was purchased from VWR life science (PA, USA).

### 2.2. Tumor cell lines and animal models

The RAW264.7 murine macrophage cell line and the B16F10 murine cell line were provided by the Cell Bank of the Chinese Academy of Sciences (Shanghai, China). The two cell lines were cultured in Dulbecco’s modified Eagle’s medium (DMEM) supplemented with 10% fetal bovine serum (FBS), 100 U/mL penicillin, and 100 µg/mL streptomycin.

Male C57BL/6 mice (aged 6–7 weeks; weighing 18–22 g) were provided by the Laboratory Animal Center of Zhejiang Chinese Medical University and all the animal experiments were performed in compliance with the protocols of the Animal Laboratory Ethical Committee of the Zhejiang Chinese Medical University. The ethical approval number for this study is IACUC-20200518-06.

### 2.3. Preparation of CA-SAL

The synthesis of SA-ODA by the carboxyl group of SA and the amino group of ODA through the action of EDC/NHS catalyst has been reported previously (She et al., 2014).

The liposomes were prepared by the ethanol injection method. CA-loaded nonmodified liposomes (CA-CL) were composed of HSPC:CHO:CA (73:22:5, W/W/W), and CA-loaded SA-modified liposomes (CA-SAL) were composed of HSPC:CHO:CA:SA (73:17:5:5, W/W/W/W). Anhydrous ethanol 10% (v/v) of the final volume of the preparation was added and dissolved in a water bath at 60 °C. After the membrane material was completely dissolved, the solution was stirred to remove most of the anhydrous ethanol. The CA solution was preheated to the same temperature was injected into the membrane and stirred in a water bath at 60 °C for 20 min to obtain the initial CA-CL and CA-SAL products. After ultrasonic dispersion treatment for 6 min (power 200 W, working for 1 s and intermittent for 1 s), the primary products were successively filtered through 0.80-, 0.45-, and 0.22-µm microporous membranes to obtain CA-CL and CA-SAL. A similar method was used to prepare DiR or Coumarin-6-loaded liposomes.

### 2.4 Characterization of CA-SAL

#### 2.4.1. Particle size distribution and zeta-potential

The average particle size, polydispersity index, and zeta potential of the prepared liposomes were determined by dynamic light scattering (Malvern, UK).

#### 2.4.2. Transmission electron microscopy

The surface morphology and particle shape were characterized by transmission electron microscope (TEM). The samples were assayed by imaging and reconstruction using the Hitachi system (Hitachi H-7650, Japan).

#### 2.4.3. Determination of encapsulation efficiency

The encapsulation rate (EE%) of CA was calculated by eliminating unencapsulated CA with a Sephadex G-50 column. After elution with distilled water, the %EE was assessed by high performance liquid chromatography (HPLC) (Agilent Co., Palo Alto, USA) after solubilization of the liposomes with 50% methanol. The %EE contents were measured from three liposome suspensions prepared separately, and expressed as mean ± SD. EE% was calculated by the formula:

$$\mathrm{EE}(\%)=\frac{W_{\mathrm{CA}}}{W_{\text{CA total}}}\times 100\%$$

W_CA_ is the amount of CA encapsulated by liposomes after removal of free CA, W_CA total_ is the total CA.

### 2.5. *In vitro* release assay

The dialysis method was used for *in vitro* drug release determination. Initially, with PBS of pH 7.4 as the release medium, 1 mL of CA solution (CA-S) and CA-SAL were placed onto a dialysis membrane (MWCO 3.5 kDa). The solutions were placed into a centrifuge tube containing 49 mL release medium and oscillated in an incubator at 37 °C. A 1 mL volume of the release medium was absorbed at the 0.5, 1, 2, 4, 8, 12, and 24 h (replenished with the equal release medium). The concentration of CA was determined by HPLC, and the cumulative release rate was calculated at each time point.

$$\text{Cumulative release rate}(\%)=\frac{C_{n}\times V_{0}+\sum C_{m}V_{m}}{C_{\mathrm{total}}\times V_{0}}$$

C_n_ is the drug concentration at t_n_, V_0_ is the volume of releasing medium, C_m_ is the drug concentration at tm, V_m_ is the amount of each sample, and C_total_ is the total drug concentration.

### 2.6. *In vitro* cytotoxicity assay

The cytotoxicity of B16F10 and RAW264.7 were assessed using the MTT cytotoxicity assay. These two cell lines were used to evaluate the cytotoxicity of CA-S and CA-SAL at different concentrations. In short, the cells were cultured in 96-well plates (3.5 × 10^4^ cells/mL) and placed in an incubator for 24 h. Next, the cells were incubated with different concentrations of CA for 24 h. MTT solution was added to each well and placed in an incubator for further incubation for 4 h. MTT solution was removed, DMSO solution was added to the precipitate, and placed on a shaker for 5 min. The absorbance was measured at 490 nm with a microplate reader (Synergy H1MFD multimode reader, BioTek Inc, winooski, northern Vermont, USA). All samples were tested in in triplicate. The cell viability (%) was calculated as follows：

$$\text{Cell viability }(\%)=\frac{{\mathrm{OD}}_{s}-{\mathrm{OD}}_{b}}{{\mathrm{OD}}_{c}-{\mathrm{OD}}_{b}}\times 100\%$$

OD_s_ represented the absorbance of the sample well, OD_c_ represented the absorbance of the control well, and OD_b_ represented the absorbance of the blank well.

### 2.7. *In vitro* cellular uptake

The cellular uptake by RAW264.7 cells was investigated using flow cytometry (Beckman CytoFlex, Brea, California, USA) by determining the fluorescence of different coumarin-6 (C6)-loaded formulations. The concentration of RAW264.7 cells was adjusted to 3 × 10^5^ cells/mL and then seeded on confocal dishes, and allowed to attached for 24 h. The original cell culture medium was replaced with fresh medium and medium-containing C6-CL and C6-SAL (50 μg/mL) and incubated at 37 °C in the dark for 4 h. The medium-treated cells were set as the negative control. The cells were washed with chilled PBS 3 times, centrifuged washed after trypsin treatment, and resuspend with PBS for later use.

For confocal laser scanning microscopy (CLSM), the concentration of RAW264.7 cells was adjusted to 3 × 10^5^ cells/mL and then seeded on confocal dishes and allowed to attached for 24 h, The original cell culture medium was replaced with medium containing C6-CL, C6-SAL, and SA + C6-SAL (50 μg/mL) and incubated at 37 °C in the dark for 4 h. The specific uptake of SA modified liposomes was determined by competitive inhibition. The cells were washed with chilled PBS 3 times and fixed with 4% paraformaldehyde for 20 min. The cells were washed with chilled PBS 3 times and then incubated with DAPI solution (5 μg/mL) for 10 min. The cells were washed and reserved. Finally, CLSM was used for observation (Zeiss LSM880, Germany).

### 2.8. *In vivo* imaging of the distribution in melanoma-bearing mice

The tumor accumulation ability of DiR-labeled liposomes in melanoma-bearing mice was observed by a noninvasive optical imaging system. DiR-S, DiR-CL, and DiR-SAL (0.60 mg/kg) were intravenously injected into B16F10 tumor-bearing mice, and the FX Pro imaging system (Xenogen, USA) was used 2, 4, 8, 12, and 24 hours after administration. After injection 24 h, tumor, heart, liver, spleen, lung, and kidney were collected, washed, and intravital fluorescence imaging was performed.

### 2.9. *In vivo* antitumor activity

C57BL/6 mice were reared in animal facilities without pathogens. A total of 1 × 10^6^ B16F10 cells/mL were injected subcutaneously into the right armpits of mice. After the tumor volume reached ∼100 mm^3^, they were randomly divided into four groups with six mice in each group as described below. For anti-PD1 Ab: The concentration of anti-PD1 Ab stock solution was adjusted to 2 mg/mL with normal saline.

1. Control (*n* = 6): PBS was injected intraperitoneally and 5% glucose was injected once every 3 days, on days 7, 10, 13, 16, and 19;
2. Anti-PD1 (*n* = 6): anti-PD1 Ab was intraperitoneally injected at the dose of 10 µg/kg once every 3 days, on days 7, 10, 13, 16, and 19;
3. CA-SAL (*n* = 6): CA-SAL was injected through the tail vein at the dose of 40 mg/kg, once every 3 days, on days 7, 10, 13, 16, and 19;
4. Combined administration (anti-PD1 + CA-SAL) (*n* = 6): anti-PD1 Ab was intraperitoneally injected with 10 µg/kg and CA-SAL was intravenously injected with 40 mg/kg, once every 3 days, on days 7, 10, 13, 16, and 19.

Tumor volume (mm^3^) was calculated as 0.5 × A × B^2^ (A: length, B: width), body weight, the inhibition of tumor growth, and tumor morphology were recorded throughout the trial, and the efficacy of each agent was comprehensively evaluated based on these data. The tumor tissue was dissected at the end of the trial for subsequent analysis of TAMs and T cells. The heart, liver, spleen, lung, kidney, and tumor were subjected to hematoxylin-eosin (H&E) staining for safety evaluation based on morphological changes of cells.

### 2.10. *In vivo* polarization of TAMs and T cell activity

Tumors were collected from the mice and minced into fine fragments in a digestion buffer containing 2% FBS and 2.5 mg/mL collagenase IV and collagenase V (Biofroxx, Guangzhou, China). The samples were incubated in the digestion buffer at 37 °C for 1 h with a shaker, filtered through a 70-μm filter, and washed twice with phosphate-buffered saline (PBS). The collected cells were stained with the following fluorescent-labeled antibodies: CD45 (Clone: 30-F11, 0058953, Biolegend, BD Biosciences, CA, USA), CD11b (Clone: M1/70, 9204453, BD Biosciences), F4/80 (Clone: BM8, 2198632, Invitrogen), CD206 (Clone: MR5D3, 0030816, Biolegend, San Diego, CA, USA), CD86 (Clone: GL1, 0316766, BD Biosciences), CD4 (Clone: GK1.5, B315274, Biolegend), CD8a (Clone: 53-6.7, C0081052418202, Tonbo, USA), and Foxp3 (Clone: 3G3, 35-5773, Tonbo). All flow cytometry was performed on a Beckman (CytoFlex, USA), and the analyses were performed using CytExpert software (CytExpert, Beckman, USA).

### 2.11. Hematoxylin–eosin staining

The heart, liver, spleen, lung, kidney, and tumor of the tumor-bearing mice were excised. The tissue blocks were trimmed to 3–5 mm^3^ with a double-sided blade and fixed with 4% paraformaldehyde. After dehydration, the tissue was embedded with paraffin and cut into a thickness of about 3 mm. Sections were stained with H&E and dehydrated. The sections were sealed with glass, and the morphology of the cells was observed under a microscope (Zeiss AXIO SCOPE.A1, Germany).

### 2.12. Statistical analysis

Values are expressed as the mean standard error (SEM) of the mean. Data were analyzed using *t*-test, one-way analysis of variance (ANOVA) using GraphPad Prism 8 software. Differences in *P* <.05 were considered significantly.

## 3. Results and discussion

### 3.1. Characterization of liposomes

**Figure 1.**
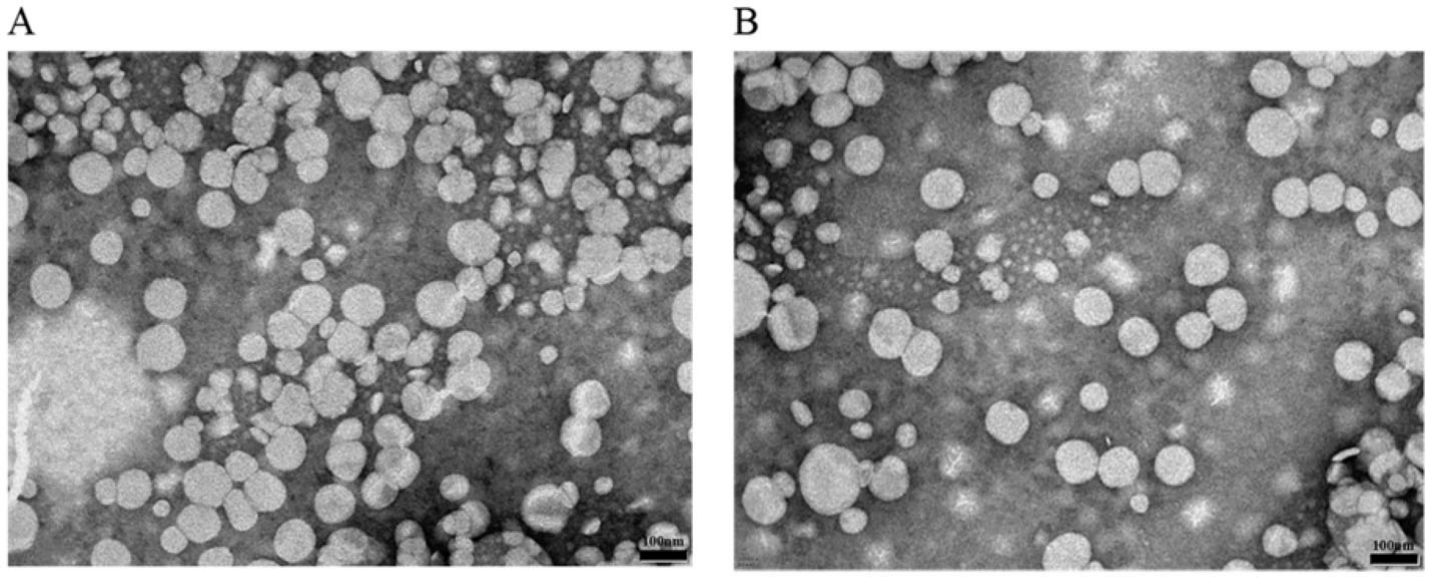
The transmission electron micrographs of (A) CA-CL and (B) CA-SAL. Note: Scale bar = 100 nm.

Many factors may affect the efficacy and toxicity of liposomal drugs. Table 1 summarizes the particle size, polydispersity index (PDI), zeta potential, and encapsulation efficiency (EE%) of liposomes. The morphology of the CA-CL and CA-SAL were further observed by TEM. The TEM images of the liposomes showed that CA-CL and CA-SAL were approximately spherical nanoparticles with an average particle size less than 100 nm (Figure 1). The particle size and zeta potential of CA-CL and CA-SAL showed that both liposomes formed spherical, homogeneous, smooth surface nanoparticles with particle size less than 100 nm, demonstrating that the decoration of liposomes with SA-ODA did not change the particle size or morphology of liposomes. The particle size of the two liposomes and the lower PDI value (<0.3), showing that the size distribution of liposomes was very narrow. The EE%, as an evaluation index of liposome quality was a key factor affecting the clinical efficacy of CA (Feng et al., 2016). The encapsulation efficiency of CA-SAL was 49.84 ± 2.96%.

**Table 1.** Characterization of liposomes (*n* = 3).

Vehicle	Particle size (nm)	PDI	zeta potential (mV)	EE%
CA-CL	87.00 ± 1.29	0.251 ± 0.005	14.6 ± 0.6	51.47 ± 3.08%
CA-SAL	90.36 ± 0.54	0.254 ± 0.006	13.8 ± 0.1	49.84 ± 2.96%

### 3.2. *In vitro* release assay

**Figure 2.**
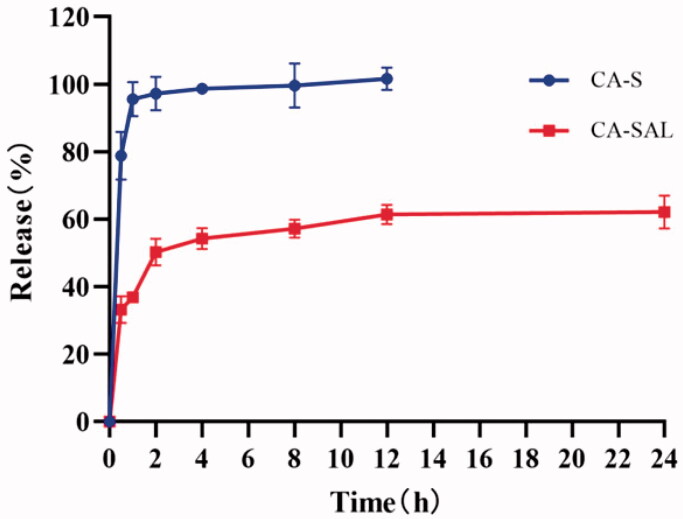
In vitro release curves of CA-S and CA-SAL. The values are shown as mean ± SEM (*n* = 3).

The release of CA-S reached 78.82% at 0.5 h, and the cumulative release rate was 98.70% at 8 h. The cumulative release rate of CA-SAL was 33.22% at 0.5 h and 62.20% at 24 h. Therefore, compared with CA-S, CA-SAL has the slow-release effect (Figure 2). The release of liposomal-encased CA was slower than that of free CA, indicating that liposomal-encased CA achieved the continuous release of CA in the blood circulation.

### 3.3. *In vitro* cytotoxicity assay

To assess the cytotoxicity of CA on B16F10 and RAW264.7 cells, cells were treated with different concentrations of CA, and cell viability was detected using the MTT assay. Studies have reported that CA inhibited tumor growth by exerting direct toxic effects on tumor cells (Zhang et al., 2019). For example, in hepatocellular carcinoma, osteosarcoma, and lung cancer, the effective concentrations of CA ranged 25–500 µM (Hou et al., 2017; Yan et al., 2017; Yamagata et al., 2018). However, the study by Li et al. showed that even at a concentration of 1000 μM, CA still could not produce significant toxicity in melanoma cells (Li et al., 2014). In addition, there have also been reports in the literature that CA could also enhance the antitumor immune effect by promoting the differentiation of M2-TAMs to M1-TAMs (Zhang et al., 2020).

**Figure 3.**
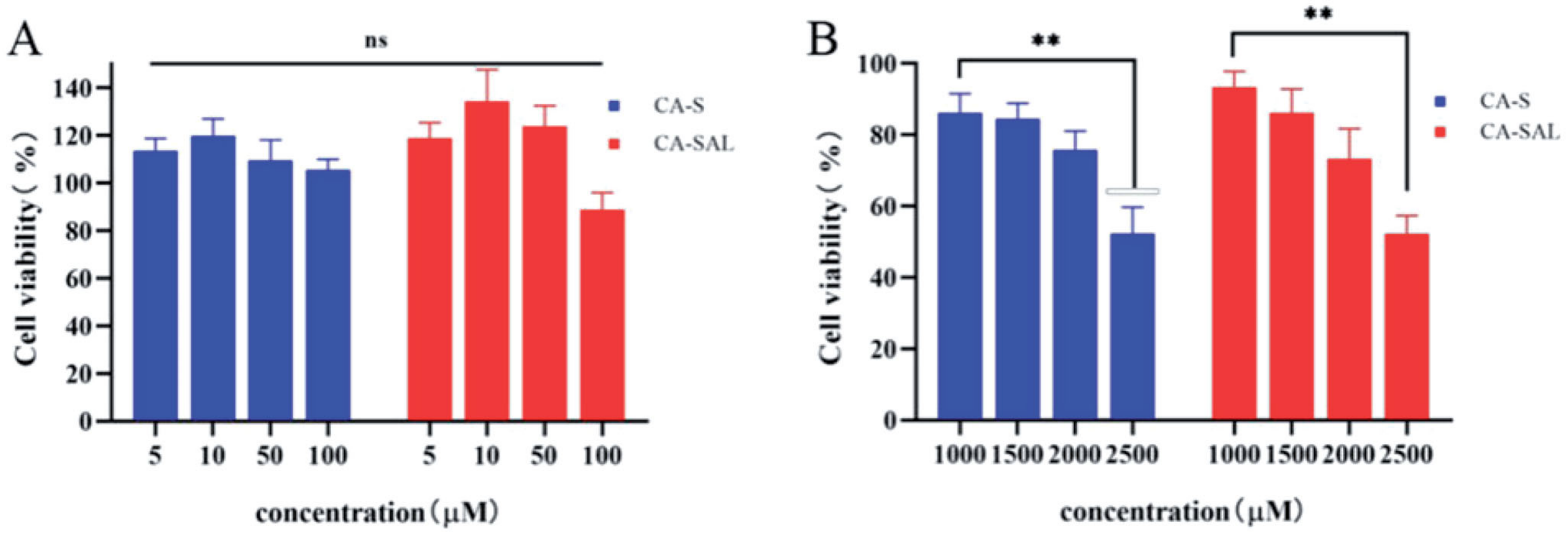
In vitro cytotoxicity assay. (A) In vitro cytotoxicity of CA-S and CA-SAL in RAW264.7. (B) In vitro cytotoxicity of CA-S and CA-SAL in B16F10 (*P < .05, **P <.01). The values are shown as mean ± SEM (*n* = 6).

The cytotoxicity of CA in B16F10 cells was further investigated. It was found that CA had an obvious inhibitory effect on B16F10 cells only when the concentration of CA was 2500 μM (*P* <.05), although it is not practical to use such concentration in the clinic (Figure 3(A)). The survival rate of both types of cells was close to 100% in CA solution and liposomes with a concentration of 5–100 μM, indicating that the toxicity of CA to both types of cells was not significant within the effective range of CA (Figure 3(B)). Therefore, CA may inhibit the growth of B16F10 melanoma mainly by regulating the polarization of TAMs.

### 3.4. *In vitro* cellular uptake

**Figure 4.**
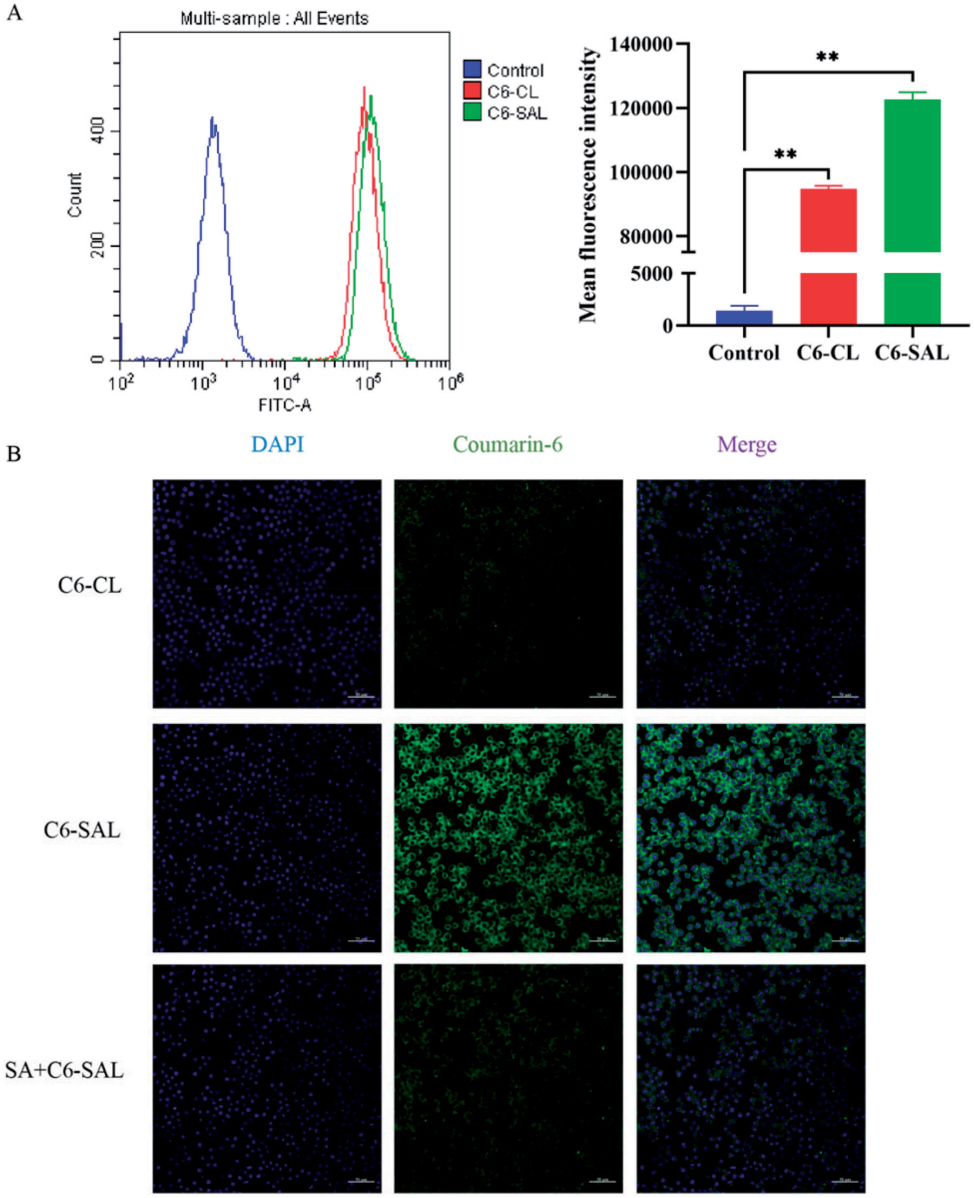
In vitro cellular uptake. (A) Flow cytometry analysis of the RAW264.7 cells treated with C6-CL and C6-SAL. (B) Confocal images of the RAW264.7 cells incubated with C6-CL, C6-SAL and SA + C6-SAL (*P < .05, **P <.01, vs control group). Note: Scale bar = 50 μm.

Flow cytometry was used to detect the cellular uptake characteristics of coumarin-6 liposomes in RAW264.7 cells. C6 was as used as a fluorescence marker. RAW264.7 cells were used as a TAM model for evaluating cellular uptake of coumarin-6-loaded liposomes by TAMs *in vitro*. SA-ODA improved the efficiency of RAW264.7 cellular uptake, and the fluorescence intensity of C6-SAL was significantly higher than that of CA-CL (*P* < .05) (Figure 4(A)).

Furthermore, CLSM was used to qualitatively test the TAMs uptake capacity of liposomes. The liposomes loaded with coumarin-6 were stained green, and the nuclei were identified by DAPI staining (blue). The fluorescence intensity of C6-SAL in cells was stronger than that of C6-CL, which confirmed the flow cytometry findings (Figure 4(B)). The fluorescence intensity of the SA + C6-SAL group was lower than that of the C6-SAL group when free SA competing receptors were added, which was due to the free SA competing receptors reducing the uptake of C6-SAL by RAW264.7 cells. This may also indicate that SA-modified liposomes had stronger targeting ability. These data demonstrated that the addition of SA on liposomes could improve the specific phagocytosis of macrophages in the preparation. In summary, SA-modified liposomes improved the specific uptake of liposomes by RAW264.7 cells. Thus, the high expression of SA receptors on the surface of TAMs (Zhou et al., 2017), may directly interact with SA modified on the surface of liposomes.

### 3.5. *In vivo* fluorescence imaging studies

**Figure 5.**
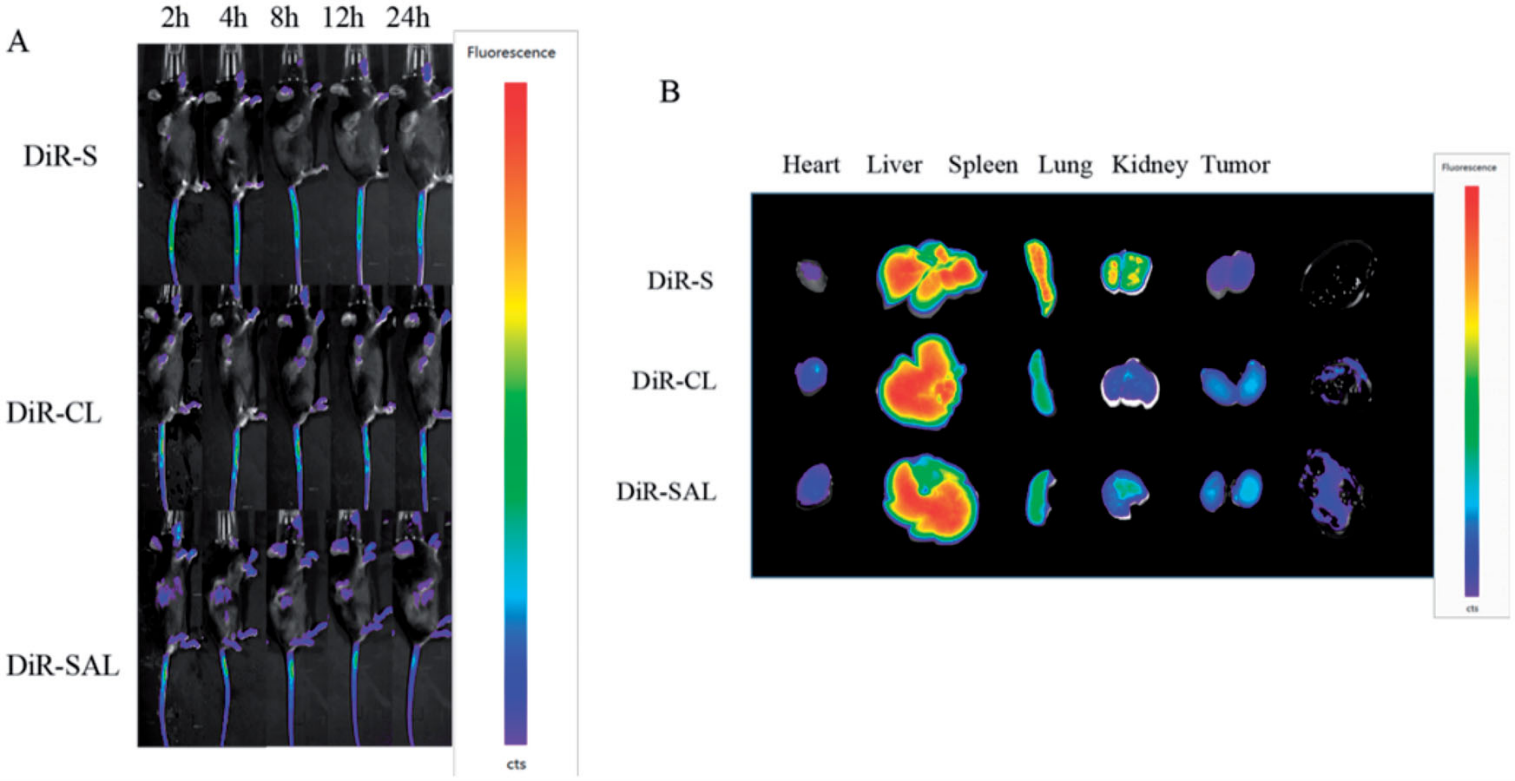
In vivo biodistribution of DiR-loaded liposomes. (A) Fluorescence imaging at different time points after intravenous DiR-loaded liposomes injection in C57BL/6 mice bearing B16F10 cells. (B) *In vivo* fluorescence images of excised organs and tumors at 24-h postinjection of DiR-loaded liposomes.

To evaluate the TAMs-targeting ability of CA-SAL *in vivo*, the tissue biodistribution of DiR-loaded liposomes in B16F10 melanoma mice was determined. After drug injection, at each observation time point, in B16F10 tumor-bearing mice treated with DiR-S, it was difficult to observe drug accumulation in the tumor. The fluorescence intensity of tumor site in mice injected with DiR-CL and DiR-SAL was significantly higher than that in the mice injected with DiR-S. The tumor fluorescence intensity of DiR-SAL injection was stronger than that of DiR-CL, which suggested that the liposomes modified with SA had stronger targeting ability (Figure 5(A)). The fluorescent signal of liposomal DiR could be maintained for up to 24 hours. To image the accumulation of DiR in different organs, mice were euthanized 24 hours after administration, and fluorescence imaging of the heart, liver, spleen, lung kidney, and tumor was performed *in vitro* (Figure 5(B)). The cumulative amount of drug in tumors of DiR-SAL-treated mice was more than that of DiR-S and DiR-CL. This may be attributed to the binding of SA to the over-expressed receptors on TAMs and then delivering SA-modified liposomes to tumor tissues for targeted effects (Jayant et al., 2007). Therefore, we used CA-SAL to improve the targeting and anti-tumor effects of CA on TAMs.

### 3.6. Antitumor activity assessment *in vivo*

**Figure 6.**
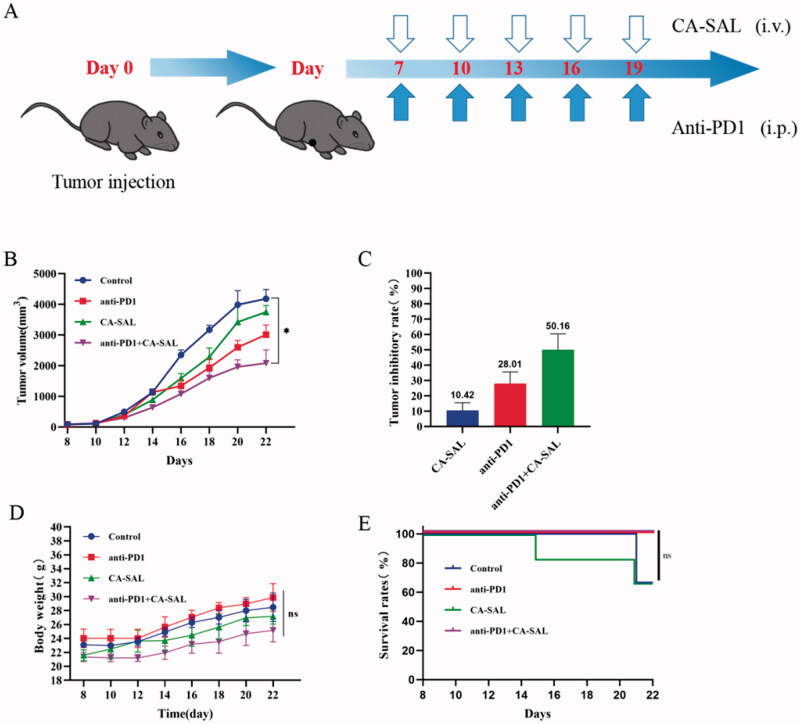
Antitumor activity assessment *in vivo*. (A) Illustration of the design of the experiments. (B) Tumor volume change curve of tumor-bearing mice (*p < .05, vs control group). (C) Tumor inhibitions rate of tumor-bearing mice. (D) Body weight curve of tumor-bearing mice. (E) Survival rate of the tumor-bearing mice receiving different treatment. The values are shown as mean ± SEM (*n* = 4–6).

After drug treatment, the tumor inhibitions rate of CA-SAL, anti-PD1 Ab, and anti-PD1 + CA-SAL were 10.42%, 28.01%, and 50.16% (Figure 6(C)). Anti-PD1 Ab and anti-PD1 + CA-SAL mice showed a marked tumor growth inhibitory effect (*P* < .05) (Figure 6(B)). The body weight among all groups was not statistically different, suggesting that there was no overt toxicity (Figure 6(D)). Before euthanasia on day 22 of the trial, the survival rate of the anti-PD1 Ab group and the combination treatment group was 100% (Figure 6(E)). Together, these results demonstrated that the combination therapy was more effective than monotherapy, and CA-SAL enhanced the therapeutic benefit of PD-1 blockade.

### 3.7. *In vivo* polarization of TAMs

The above *in vitro* cytotoxicity study results indicated that CA-SAL did not exert any cytotoxicity in the mouse macrophage cell line RAW264.7, CA had a limited cytotoxic effects on B16F10 tumor cells when the concentration was below 2500 μM. Thus, we evaluated whether the therapeutic effects of CA-SAL and anti-PD1 Ab on tumors mainly stemmed from the immunomodulatory ability of CA to TAMs. The polarization ability of CA-SAL *in vivo* was evaluated by analyzing the proportion of M1/M2 subtype macrophages.

**Figure 7.**
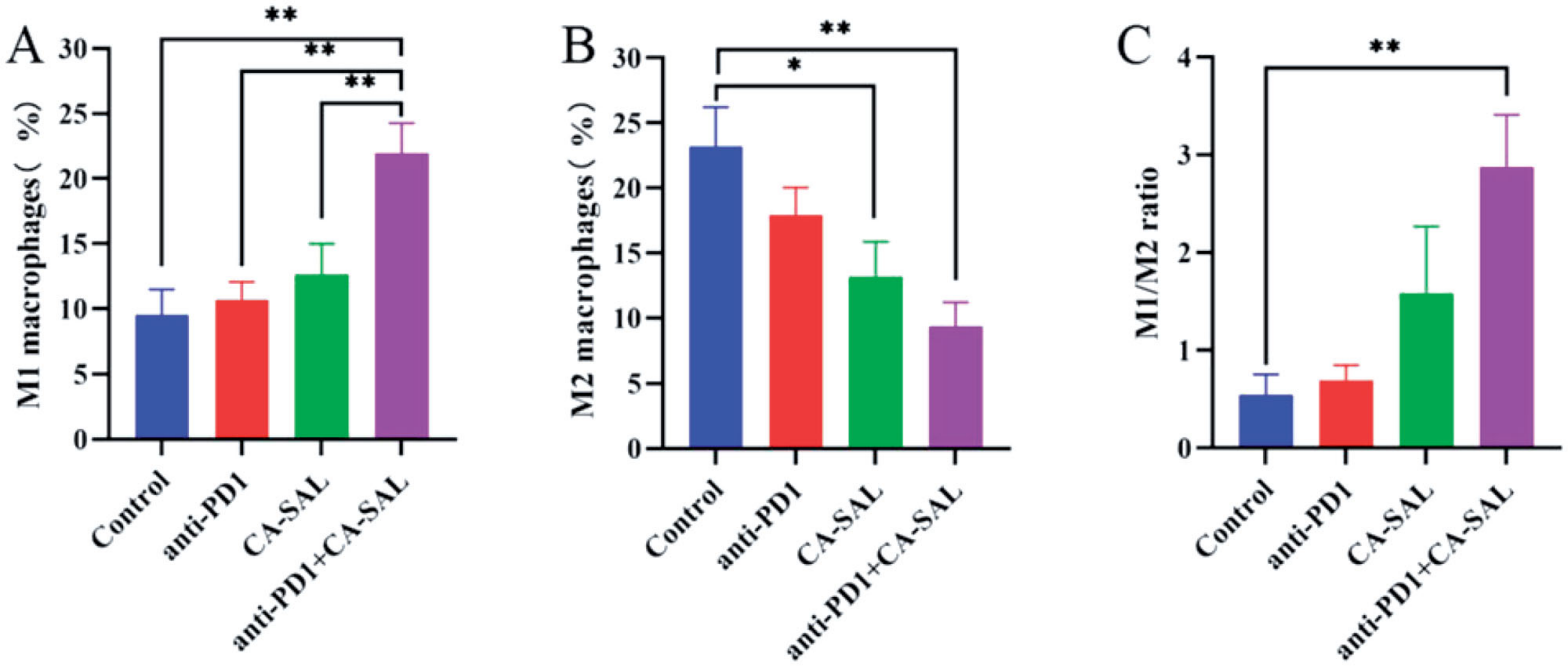
The *in vivo* polarizability of TAMs in the administration group was assessed by analyzing the proportion of M1/M2 subtype macrophages in the tumor tissue. (A) The proportion of M1-TAMs (CD45^+^F4/80^+^CD11b^+^CD86^+^) (**p < .01, *p < .05, vs anti-PD1 + CA-SAL). (B) The proportion of M2-TAMs (CD45^+^F4/80^+^CD11b^+^CD206^+^) (**p <.01, *p < .05, vs control group). (C) The ratio of M1/M2 TAMs (**p < .01, *p <.05, vs control group). The values are shown as mean ± SEM (*n* = 4–6).

Currently, a growing number of studies have found that the response to anti-PD1 Ab therapy and other immunotherapies was dependent on TME (Sharma and Allison, 2015). Macrophages are the dominating immune cell population in the TME. TAMs play a double-edged sword role in tumors, showing both the anti-tumor M1 phenotype and pre-tumoral M2 phenotype and possess the ability of interphenotypic transformation. Our results showed that the proportion of M2 TAMs in the combination treatment group of CA-SAL and anti-PD1 + CA-SAL was significantly reduced (*P* < .05), and the proportion in the anti-PD1 Ab group was also decreased, albeit not significantly (Figure 7(B)). Only the combination treatment group presented a significant increase in the proportion of M1-TAMs (Figure 7(A)). To further demonstrate the ability of each group to regulate the TAM phenotype, we analyzed the proportion of M1/M2 subtypes of macrophages, the increase in the M1/M2 ratio could indicate an improvement in the polarization effect. The M1/M2 of the combination treatment group was significantly higher than that of the other groups (*P* <.05), indicating that the combination treatment group could effectively repolarize M2-TAMs into M1-TAMs (Figure 7(C)). Thus, CA inhibited the growth of B16F10 melanoma by promoting the polarization of M2 to M1-TAMs. Therefore, the reshaping of TAMs was associated with anti-tumor effectiveness, which was consistent with the results of tumor inhibition in each group.

### 3.8 T-cell activity *in vivo*

**Figure 8.**
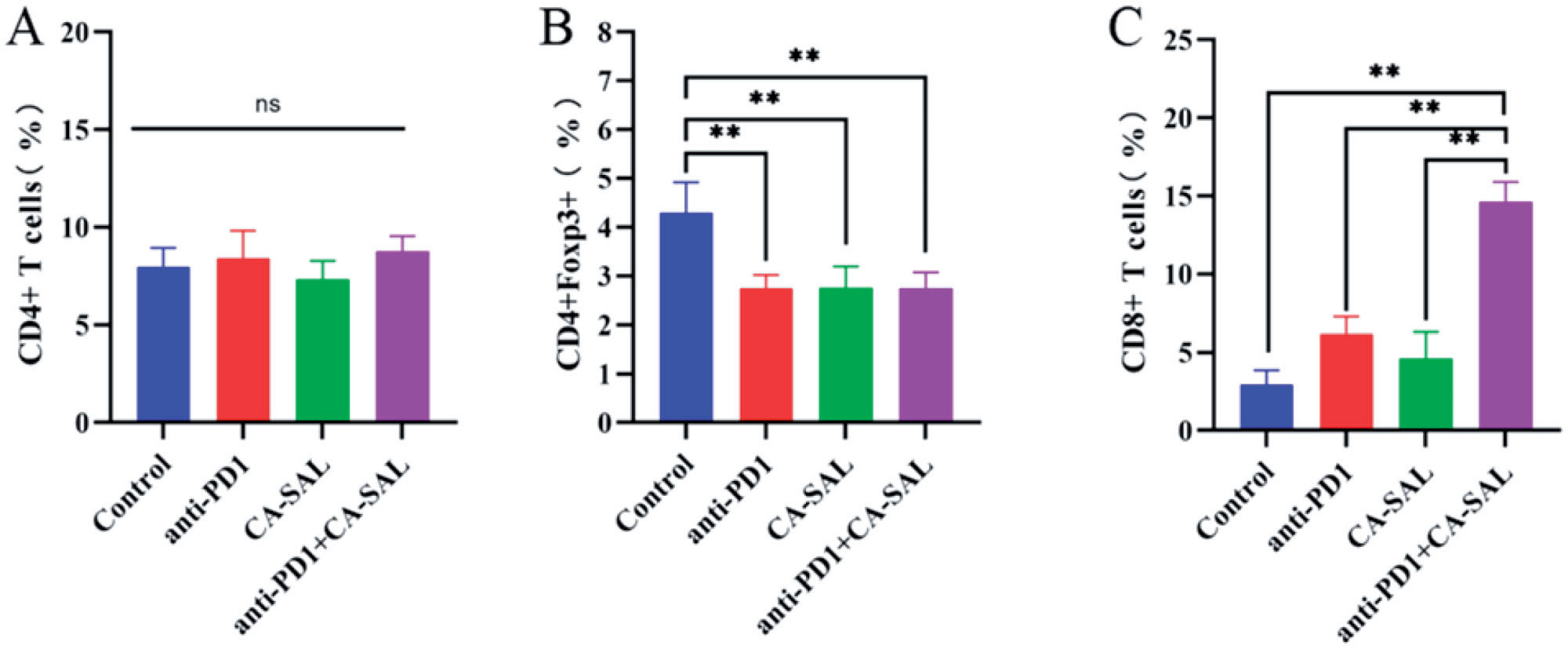
The activity of T cells in the administration group was assessed by analyzing the proportion of CD4+ and CD8 + T cells in the tumor tissue. (A) The proportion of CD4 + T cells. (B) The proportion of CD4 + Foxp3 + T cells (**p <.01, *p <.05, vs control group). (C) The proportion of CD8 + T cells (**p < .01, *p < .05, vs anti-PD1 + CA-SAL). The values are shown as mean ± SEM (*n* = 4–6).

To further investigate whether combination therapy could enhance immune response, the percentages of CD4^+^, CD8^+^, and CD4^+^Foxp3^+^ in T cells were analyzed by flow cytometry. There was no significant change in the proportion of CD4^+^T cells in each group (Figure 8(A)). The proportion of CD4^+^Foxp3^+^ was significantly reduced across all treatment groups (*P* <.05) (Figure 8(B)). However, the proportion of CD8^+^T cells was increased after anti-PD1 Ab therapy and the combination of CA-SAL and anti-PD1 Ab therapy (*P* < .05) (Figure 8(C)).

T-cell activation is the main mechanism involved in the response to anti-PD1 Ab treatment. In different models, malignant tumors recruit regulatory T cells (CD4^+^Foxp3^+^T cells) to suppress the CD8^+^T cell response and maintain immune tolerance, which suppresses the antitumor immune response and promotes tumor progression (Xiong et al., 2019). CD8^+^T cells are the key to kill tumor cells, and their presence inhibits tumor immune escape mechanisms, improves immune activity, and plays an important role in anti-tumor efficacy (Lu et al., 2019). PD-1 blockade therapy has been shown to enhance the activity of CD8^+^T cells and has demonstrated clinical benefits in a variety of cancer types (Taube et al., 2014; Fang et al., 2019). Our study found that the immune cell phenotyping was altered in the TME of the tumor following treatment. It was worth noted that both combination therapy and monotherapy reduced the proportion of CD4^+^Foxp3 ^+^ T cells. Meanwhile combination therapy significantly increased the proportion of CD8^+^T cells (*P* < 0.05), compared with other groups. Anti-PD1 Ab was effectively briefly bound to PD-1+ tumor-infiltrating CD8^+^T cells in the early stages after administration. This may be rapidly followed by PD-1-TAMs capture of the anti-PD1 Ab from the surface of the T cell, which thereby reduces its effectiveness (Arlauckas et al., 2017). The proportion of CD8 ^+^ T cells following combination therapy was higher than that of the single anti-PD1 Ab group, which may indicate that with the polarization of TAMs by CA-SAL, anti-PD1 Ab could bind to fewer TAMs and act more on T cells. In other words, in the combination treatment group, the reduction of M2-TAM could increase the effects of anti-PD1 Ab on T cells, thereby activating T-cell function. Thus, when CA-SAL are used in combination with anti-PD1 Ab, therapies targeting tumor macrophages may gain additional benefit by increasing immune checkpoint-blocking drug delivery to CD8^+^T cells, thereby enhancing immunotherapeutic activity.

### 3.9. Hematoxylin–eosin staining

**Figure 9.**
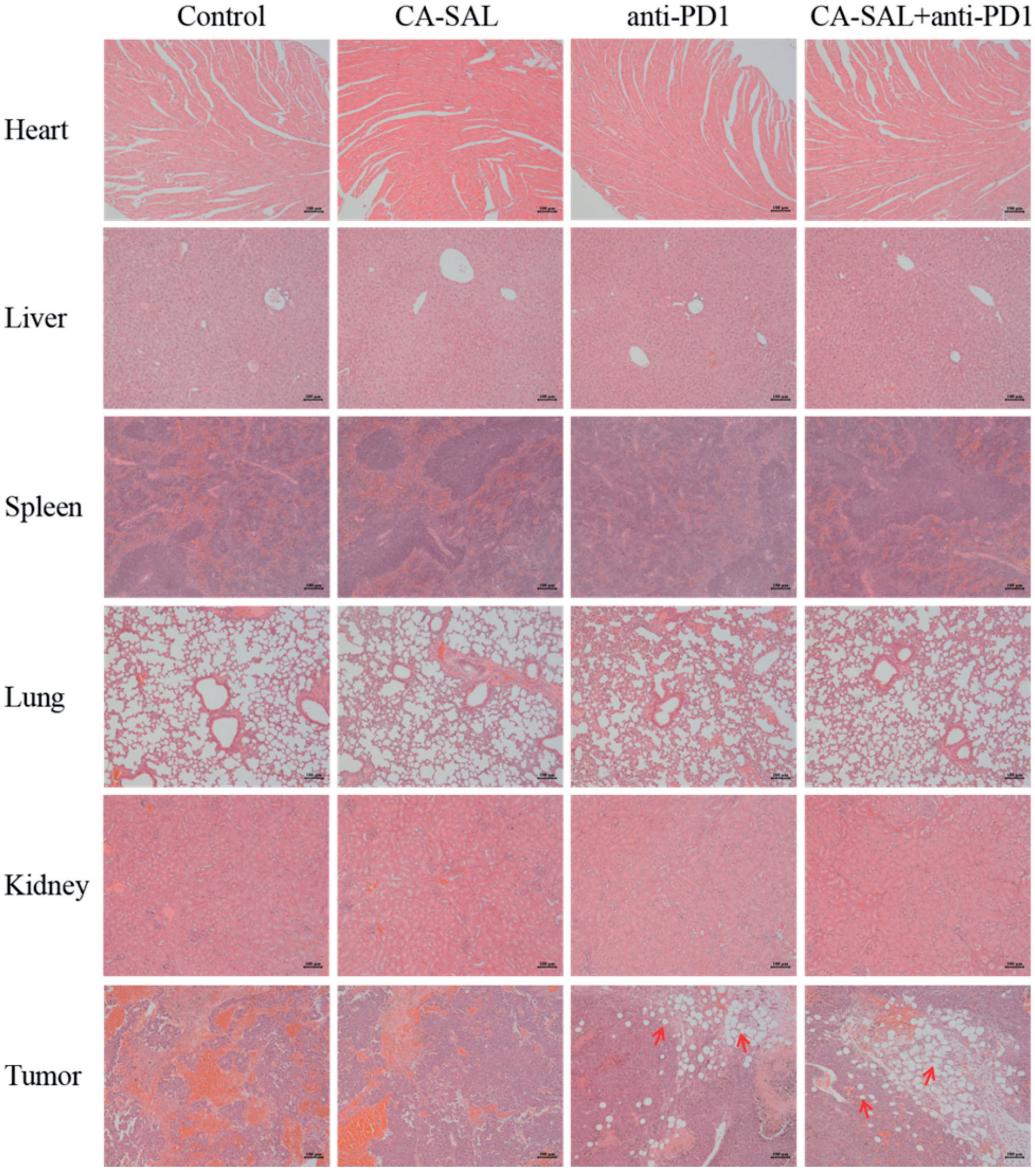
Histopathology of heart, liver, spleen, lung, kidney and tumor sections with hematoxylin and eosin (H&E) staining of different experimental groups. Note: Scale bar = 100 μm.

Malignant melanoma cells proliferated in both the control group and the CA-SAL group (Figure 9). Large areas of tumor tissue necrosis were observed in the anti-PD1 Ab and combination treatment groups; although, the morphology and structure of the heart, liver, spleen, lung, and kidney were not adversely affected. These results indicated that combination therapy could not only significantly inhibit the growth of malignant melanoma but was also relatively safe.

Overall, CA-SAL could increase the proportion of M1, reduce the proportion of M2, and reshape the TME. To some extent, it could also increase binding between anti-PD1 Ab and CD8^+^T cells. At the same time, the anti-PD1 Ab directly acted on CD8^+^T cells, increased the proportion of CD8^+^T cells and enhanced anti-tumor activity. Therefore, combination therapy of CA-SAL and anti-PD1 Ab could effectively target TAMs and repolarize M2-TAMs to M1-TAMs and improve the activity of CD8^+^T cells, exerting a powerful anti-tumor effect.

## 4. Conclusions

In this study, we designed CA-SAL preparation consisting of a targeted liposome modified with SA, and evaluated CA-SAL and its combination with anti-PD1 Ab for the treatment of melanoma. The *in vivo* administration of CA-SAL promoted the uptake and continued release of CA which enhanced its therapeutic efficacy. The combination therapy could activate the T-cell immune response by increasing the proportion of CD8^+^T cells and M1-TAMs, thereby controlling growth and progression of B16F10 melanoma cells. Our findings indicate that the combination of CA-SAL and anti-PD1 Ab can be considered a potential therapeutic approach to enhance melanoma immunotherapy.
